# Backlash policy diffusion to populists in power

**DOI:** 10.1371/journal.pone.0273951

**Published:** 2022-09-23

**Authors:** James F. Adams, Tobias Böhmelt, Lawrence Ezrow, Petra Schleiter

**Affiliations:** 1 UC Davis, Davis, CA, United States of America; 2 University of Essex, Colchester, United Kingdom; 3 University of Oxford, Oxford, United Kingdom; University of Glasgow, UNITED KINGDOM

## Abstract

We analyze how parties respond programmatically to populist parties in power abroad. Political parties often copy the policies of governing parties in other countries–a phenomenon that contributes to waves of transnational policy diffusion. We report the first large-scale comparative study showing that populist parties in government abroad trigger the opposite reaction: instead of inspiring emulation, their highly visible governing dilemmas provoke a policy backlash by parties in other states. We argue that dilemmas arise because populist parties confront unique and debilitating trade-offs between maintaining their anti-system posture and governing effectively, which make them electorally vulnerable. Other parties observe foreign populists’ governing dilemmas and respond by distancing themselves in order to avoid these problems. We detect this “foreign populist backlash effect” using spatial econometric analysis, a method that allows us to estimate international policy connections between parties, applied to over 200 European parties’ programmatic positions since the 1970s. Our findings illuminate parties’ election strategies and show that this backlash effect constrains the spread of populism across Western democracies.

## Introduction

The rise of populism has received considerable attention [1]. Does the rise of populist parties to government further fuel the spread of their policies to other parties abroad? Conventional wisdom suggests that the success of populist parties aids the spread of their policies, with prior work examining how populist challenger parties’ rise in the *electoral arena* influences domestic party competition [2], mainstream party policy positions [3, 4], and the cross-national growth of the populist right [5]. Here, for the first time, we explore systematically how the ultimate success of populist parties–defined by their ascent to the *government arena* [6, 7]–affects the cross-national spread of their policies to parties abroad, a question that remains surprisingly understudied given scholars’ attention to the impact of rising populism on politics around the world.

When parties gain power, their policy programs are often copied and emulated in other countries. Cases in point include the spread of neo-liberal economic programs [8] or “Third Way” social democratic policies [9]. Other parties’ policy stances help party strategists navigate difficult programmatic choices because they serve as a heuristic, i.e., a cognitive shortcut to guide rational action in uncertain and complex environments [10]. In an attempt to position themselves successfully, parties copy the policies of their most visible and successful peers abroad, i.e., foreign governing parties [e.g., 8, 9, 11].

However, there is some anecdotal evidence highlighting that *populist* governing parties tend to engender a “distancing” or “backlash” reaction among parties abroad rather than policy emulation. For instance, when Martin Schulz became leader of the German Social Democrats (SPD) in 2017, he reinforced his party’s commitment to protecting equal rights, explicitly stating that he was doing so in direct opposition to Donald Trump’s populist position: “we will never give up our values, our freedom and democracy, no matter what challenges we are facing […]. That a US president wants to put up walls, is thinking aloud about torture and attacks women, religious communities, minorities, people with handicaps, artists and intellectuals with brazen and dangerous comments is a breach of taboo that’s unbearable.” And in 2018, the French President, Emmanuel Macron, positioned his party *La République en Marche* against populist, anti-immigration parties in Europe. Responding to criticism from Italy’s Matteo Salvini and Hungary’s Viktor Orban, he declared “[i]t is clear that today a strong opposition is building up between nationalists and progressives and I will yield nothing to nationalists and those who advocate hate speech.”

We propose that the troubled governing experiences of populist parties *in power* set a visible and negative precedent that inspires a policy backlash from parties in other countries. Wielding power requires populists to work within government, while their programs promise the opposite: to disrupt governments and the “corrupt” political elites that inhabit them. Once populists enter government, this stance undermines their future success. Specifically, populist parties espouse an anti-system, anti-elite worldview that asserts the inherent corruption of mainstream political parties, government bureaucracies, and experts [12]. Once in power, these parties’ programmatic commitments to disrupting the establishment generates difficulties in working successfully with coalition partners [13, 14], government bureaucracies [15], and expert advisers [16]. This undermines populists’ ability to address pressing national problems [17] and to realize their policy goals [18]. Such competence failures are electorally damaging [19]. Moreover, if populist parties moderate their anti-elitist stance to govern effectively, they undercut their core electoral appeal as disruptors of the establishment [20]. These tensions, between governing effectively and fulfilling disruptive, anti-elitist electoral promises, arise from the distinctive nature of populist programmatic commitments–shared by the populist right and left–from which there is no easy escape.

That is, populists’ core characteristics [12] create pressures for these parties between maintaining their populist posture and governing effectively. In turn, populist parties confront two unique governing challenges. First, their commitment to disruptive anti-elitism impedes their ability to work with elites, e.g., coalition partners, civil servants, and experts, to successfully address pressing national problems. As a result, populist incumbents have a poor track record addressing significant national policy challenges such as environmental issues [e.g., 16, 17]. Second, similar difficulties also compromise populist incumbents’ ability to implement electoral pledges, even in their core issue areas such as immigration and European integration. Moreover, populists tend to make more extreme policy pledges than the typical incumbent–hence, the gap between populist governing parties’ promises and actual legislative outputs can be especially large.

Governments’ failures to address pressing national challenges and to deliver on policy pledges have electoral costs. Voters may conclude from the “policy shortfall” that populist incumbents are ineffective, or–perhaps equally damaging–voters may feel betrayed, concluding that populists’ pre-election promises were insincere.

Populists’ unsuccessful governing experiences constitute a relevant and visible precedent (8) to parties in other countries. Parties observe foreign populists’ governance dilemmas and the attendant electoral penalties, and connect them to populist policy programs. Unlike other governing parties, populist incumbents experience a exceptional dilemma. They risk electoral punishment regardless of whether they work effectively within the system to deliver successful policy, thereby betraying their populist stance, or whether they maintain their disruptive anti-elitism, thereby undercutting their ability to successfully drive through their policies. The heightened media attention incumbents receive [21] enhances the visibility of these debilitating tensions to parties observing from abroad, who seek to avoid these problems by distancing their own policies. Hence, we anticipate that other parties’ reactions to foreign populist incumbents forms an exception to the tendency of cross-national party policy diffusion. Specifically, we propose a foreign governing populist backlash effect, whereby parties consciously distance their own policy programs from those of foreign left- and right-wing populist parties in government–even while emulating non-populist incumbents abroad [8].

By theorizing and offering evidence of this backlash effect against populist incumbents, our study makes several contributions. First, it informs debates about the contagion of populism. In contrast to work which suggests that populist parties’ policies diffuse quite readily, we show that populists, once in power, generate countervailing distancing reactions abroad. Second, we contribute to the literature on cross-national policy diffusion, by showing that political competition in one country can, under certain conditions, trigger a backlash (rather than emulation) by parties abroad.

### Design

To evaluate our “populist backlash” hypothesis empirically, we compiled data comprising 264 parties in 26 European democracies between 1977 and 2017. In the S10 Table in S1 Appendix, we also report results for only the post-1994 period, since most populist incumbents took office after the mid-1990s and to address concerns that the measure of party populism does not vary over time. The data set is time-series cross-sectional and, following earlier research in this context [8, 9] has the party-year as the unit of analysis. All parties are incorporated when data are available, including “niche” parties with, for example, regional or agrarian platforms. Parties enter the data with the first election in which they compete for office; they exit the sample once they no longer participate in national elections. Parties, their entry, and exit dates are based on the Comparative Manifesto Project [CMP; 22]. The total number of observations is 4,049 party-years; non-election years are interpolated. For this interpolation, we assume that party characteristics do not change until the next election year [see 8, 9 for the same approach and more details on the underlying rationale].

Our main models are based on OLS regression comprising spatial lags that are also temporally lagged [8, 23]. Dealing with problems of simultaneity bias in spatial models based on OLS can be addressed by time-lagging the spatial lag. However, this only works and deals with simultaneity bias if the errors are serially independent. While a temporally lagged dependent variable can eliminate serial correlation, which we include, there is also a theoretical argument for doing so: developing party manifestos is a time-consuming process (see below). Having said that, the S13 Table in S1 Appendix presents a spatial maximum-likelihood model, which does not assume a temporally lagged spatial lag and addresses simultaneity bias directly [24].

The CMP provides a generic left-right measure on party positions, which we use as our main dependent variable [22]. We linear-proportionately rescale the original CMP left-right variable to an interval ranging from 1 (extreme left) to 10 (extreme right). Arguably, the left-right dimension is the most important indicator for issue competition and a common vocabulary for political elites and voters. Yet, populists clearly also take positions on more focused dimensions such as immigration and multiculturalism, European integration, and economic policy, and parties may respond positively to populists on these dimensions instead. Therefore, we extend our analysis to these additional dimensions (results summarized in the S6-S8 Tables in S1 Appendix), and differentiate between the reaction of populist and non-populist receiving parties (S12 Table in S1 Appendix). To preview, these additional analyses substantiate the conclusions we report for left-right ideology below.

Our core explanatory variables are spatial lags of this left-right dependent variable [25], which allow us to estimate how foreign incumbents influence a party’s current left-right position. That is, we estimate a party’s left-right position in the current year as a function of other parties’ lagged positions. The latter are weighed via a matrix, ***W***, which specifies the relationship between parties (senders and receivers), i.e., parties that can in principle exert an influence on others. For each spatial lag, we multiply ***W*** with a temporally lagged version of our dependent variable. The temporal lag we employ uses positions of potentially influential parties in the year before their last election. That is, we predict a focal domestic party’s position in the current year as a function of the foreign incumbents’ positions from the year before their last election. For instance, parties competing in the 2002 Dutch national election would react to the governing British Labour Party’s position in 1997. This lag structure reflects the fact that developing party manifestos is a “time-consuming process […] which typically takes place over a two-three year period during which party-affiliated research departments and committees draft sections of this manuscript, which are then circulated for revisions and approval upward to party elites and downward to activists” [26: 832]. As a result, instantaneous effects are unlikely, and we use parties’ positions from the year before the last election in their country when constructing the spatial lags. This approach has the merit of following the lag structure employed in prior work on positive learning by parties from successful precedents [8, 9], which ensures that our study–the first to hypothesize a negative backlash reaction to the troubling precedent set by populist incumbents–is directly comparable.

We specify two different weighting matrices to create two different spatial lags for (non-) populists in power abroad. First, there is the populist matrix where entries are set to 1 only if a sender party j is defined as a populist party that does not compete for office in the same country as the receiver party i, and j has been recently in power (either governing alone or in a coalition at some point in the last year before the most recent election in their country). This leads to the first spatial variable, ***W***_*y*_^*Populist Incumbent*^, which captures the lagged left-right positions of all populist incumbents abroad as a potential influence on parties “at home.” For the populist-party matrix, the impact of all non-populist parties is set to 0. Populist parties are defined according to the Populism and Political Parties Expert Survey (POPPA) by (27). This data set covers up to 250 political parties in 28 European countries that were represented in parliament in 2017/2018. The POPPA data are time-invariant and, thus, we assume that parties’ general populist ideology does not change over time, which makes it possible to use the POPPA data for years before 2017. In the S1 Appendix, we address potential concerns about this time-invariant treatment of populist parties, for instance the claim that the Swiss People’s Party or the Austrian FPÖ were not populist prior to the mid-1990s, by constraining our sample to the post-1994 period only (S10 Table in S1 Appendix). In addition, we re-estimate our main model using the time-variant PopuList data set. Both robustness checks increase the confidence in the results we present below. Parties that are initially covered by the CMP data, but no longer exist in 2017/2018 are omitted from the analysis as they are not coded in the POPPA data. As discussed in [27], POPPA outperforms other data sources in terms of accuracy, validity, and coverage.

The scale that is used to classify parties as populist is based on factor regression scores of five dimensions of populism and ranges between 0 (not at all populist) and 10 (very populist). Specifically, the five dimensions that the Populism Score comprises are: a Manichean discourse (parties see politics as a moral struggle between good and bad), the indivisibility of people (parties consider the ordinary people to be indivisible, i.e., homogeneous), the general will of the people (parties consider the ordinary people’s interests to be singular), people centrism (parties believe that sovereignty should lie exclusively with the ordinary people), and anti-elitism. We define parties as populist if they achieve a populism score higher than 5. Data for incumbency are taken from [28]. Our data sample comprises 4,049 party-years, of which only 1,405 pertain to incumbents. A populism score cut-off point of 5 yields 461 populist incumbent-years generated by 16 populist parties that were in government at some point during our observation period. These parties are: True Finns (Finland), Communist Party (France), Go Italy (Italy), League (Italy), Coalition of the Radical Left (Greece), Austrian Freedom Party (Austria), Progressive Party of the Working People (Cyprus), United Democratic Union of Cyprus (Cyprus), Democratic Party (Cyprus), ANO 2011 (Czech Republic), Alliance of Federation of Young Democrats (Hungary), Law and Justice (Poland), Social Democratic Party (Slovenia), Slovenian Democratic Party (Slovenia), New Slovenian Christian People’s Party (Slovenia), and the Democratic Party of Pensioners of Slovenia. In S9 Table in S1 Appendix, we assess the robustness of our main result when altering the cut-off point for defining populist parties via the Populism Score variable.

Our second main spatial variable, ***W***_*y*_^*Non-Populist Incumbent*^, is based on a matrix that mirrors the first one, but focuses on non-populist incumbents abroad. That is, entries in this matrix are set to 1 only if a sender party j is defined as non-populist, does not compete for office in the same country as the receiver party i, and has been recently in power. The data sources for creating this matrix are the same as for the first matrix above, with non-populist parties defined as those scoring at or below 5 on the 0–10 populism factor variable from the POPPA data set. This second matrix is used to create a second spatial lag for (non-) populists in power abroad, capturing the left-right positions of non-populist incumbents in other countries.

The matrices are not row-standardized. Row standardization would imply that the number of parties abroad at a given time is less relevant to programmatic diffusion. However, party strategists should only consider other parties’ positions if they expect the marginal value of the information gathered to exceed the marginal cost of obtaining it, which is not consistent with the allocation of a fixed amount of effort [8, 9, 23, 29]. In other words, as the number of foreign parties increases, diffusion effects are likely greater, but with a diminishing marginal effect of each additional party.

We account for any biases stemming from common exposure or alternative exogenous factors [25, 30] and include the (one-year) temporally lagged dependent variable, party-fixed effects, and year-fixed effects. In addition, we control for domestic influences shaping parties’ policy positions and party policy diffusion [e.g., 26, 29]. First, we control for the influence of domestic parties by creating a spatial lag called ***W***_*y*_^*Domestic*^, where matrix entries only receive a value of 1 if i and j are different parties competing in the same political system (otherwise 0). While previous research shows that parties react differently to rival populist and non-populist parties [31, 32], we are merely interested in controlling for a general diffusion effect at the domestic level, which ***W***_*y*_^*Domestic*^ is sufficient for.

Second, we control for the influence of the median voter [6] using Eurobarometer data on respondents’ left-right self-placement on a scale of 1 (left) to 10 (right) and employ Tukey’s method to calculate final values from the individual level data. Third, Ward et al. [33] argue that the effects of economic globalization are conditioned by the position of the median voter. We thus control for the economic component of the KOF Globalization Index [34] and include the multiplicative interaction Median Voter * Economic Globalization.

Finally, in some models, we include a dichotomous variable for whether focal (receiver) parties were in government in the year before their last election. We use the data in [28] for this binary variable. Government and opposition parties likely react differently to policy influences from abroad [8]. The former have recently been electorally successful themselves, while the latter have to position their manifestos to regain political power. For instance [35], argue that government parties take more radical positions to compensate for the dilution of their ideology, while opposition parties moderate their policy to appear “fit to govern.” Hence, parties’ incentives to distance themselves from populists abroad may differ for governing parties versus those in opposition. We account for this possibility via an interaction with the core spatial variables.

## Results

Table 1 reports our main results, where the dependent variable is the left-right position of a receiving party according to the CMP measure. The first model analyzes how receiving parties react to foreign populist incumbents’ left-right positions (the variable ***Wy***^*Populist Incumbent*^). Model 2 estimates the influence of foreign non-populist incumbent parties’ lagged left-right positions (the variable ***Wy***^*Non-Populist Incumbent*^) replicating previous research, which shows that parties learn from foreign non-populist incumbents [8, 9]. The third model estimates how domestic parties react to both populist and non-populist incumbents’ positions abroad, while considering an indicator denoting whether the receiving party was in government, which is interacted with the ***Wy***^*Non-Populist Incumbent*^ and ***Wy***^*Populist Incumbent*^ variables. Governing and opposition parties may react differently to populists in office abroad [8, 35].

**Table 1:** Party policy diffusion–Populist backlash.

	Foreign Populist Incumbents’ (1)	Foreign Non-Populist Incumbents’ (2)	All Foreign Incumbents’ (3)
Lagged Party Position	0.779	0.779	0.778
	(0.010)***	(0.010)***	(0.010)***
Lagged Median Voter	0.182	0.217	0.199
	(0.114)	(0.113)*	(0.114)*
Lagged Economic Globalization	0.012	0.016	0.014
	(0.008)	(0.008)**	(0.008)*
Lag Median Voter *	-0.002	-0.003	-0.003
Lagged Economic Globalization	(0.002)	(0.002)**	(0.002)
Incumbent			-0.114
			(0.005)**
**Wy** ^Domestic^	0.003	0.003	0.003
	(0.001)***	(0.001)***	(0.001)***
**Wy** ^Populist Incumbent^	-0.013		-0.010
	(0.004)***		(0.004)**
**Wy** ^Non-Populist Incumbent^		0.003	0.002
		(0.002)**	(0.002)
**Wy**^Populist Incumbent^ * Incumbent			-0.003
			(0.001)**
**Wy**^Non-Populist Incumbent^ * Incumbent			0.001
			(0.000)***
Observations	4,049	4,049	4,049
Year and Party Fes	Yes	Yes	Yes
R^2^	0.872	0.872	0.873

*Notes*. Table entries are coefficients (and standard errors in parentheses). The constant and year- and party-fixed effects are included

in all models, but omitted from the presentation. All explanatory variables are lagged one year. The spatial lags are calculated based on parties’ positions from the year before their last national election.

* p<0.10

** p<0.05

*** p<0.01

**Fig 1:**
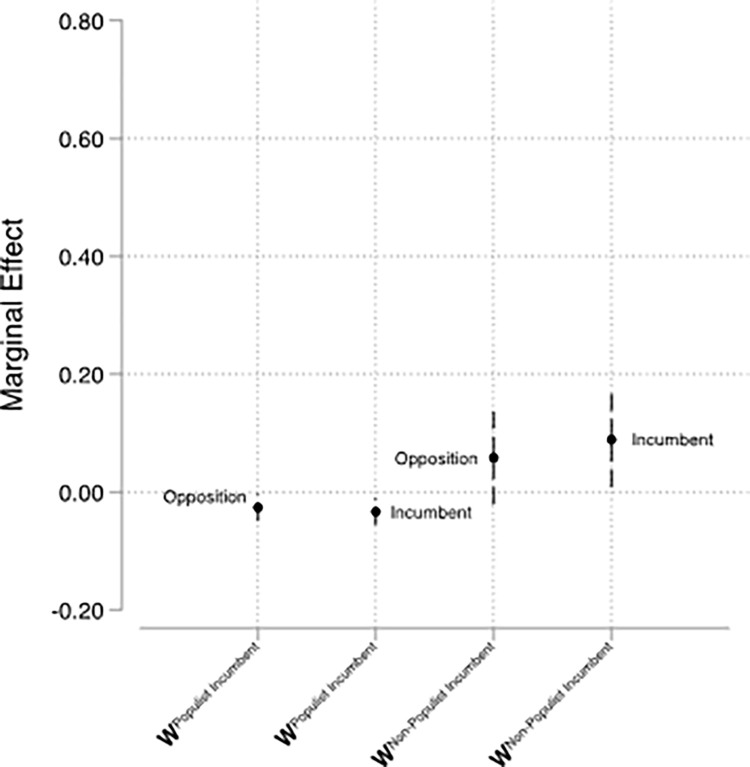
Short-term effects. *Notes*. Point estimates pertain to average marginal effects. Dashed lines signify 95 percent confidence intervals. Estimates based on Model 3.

**Fig 2:**
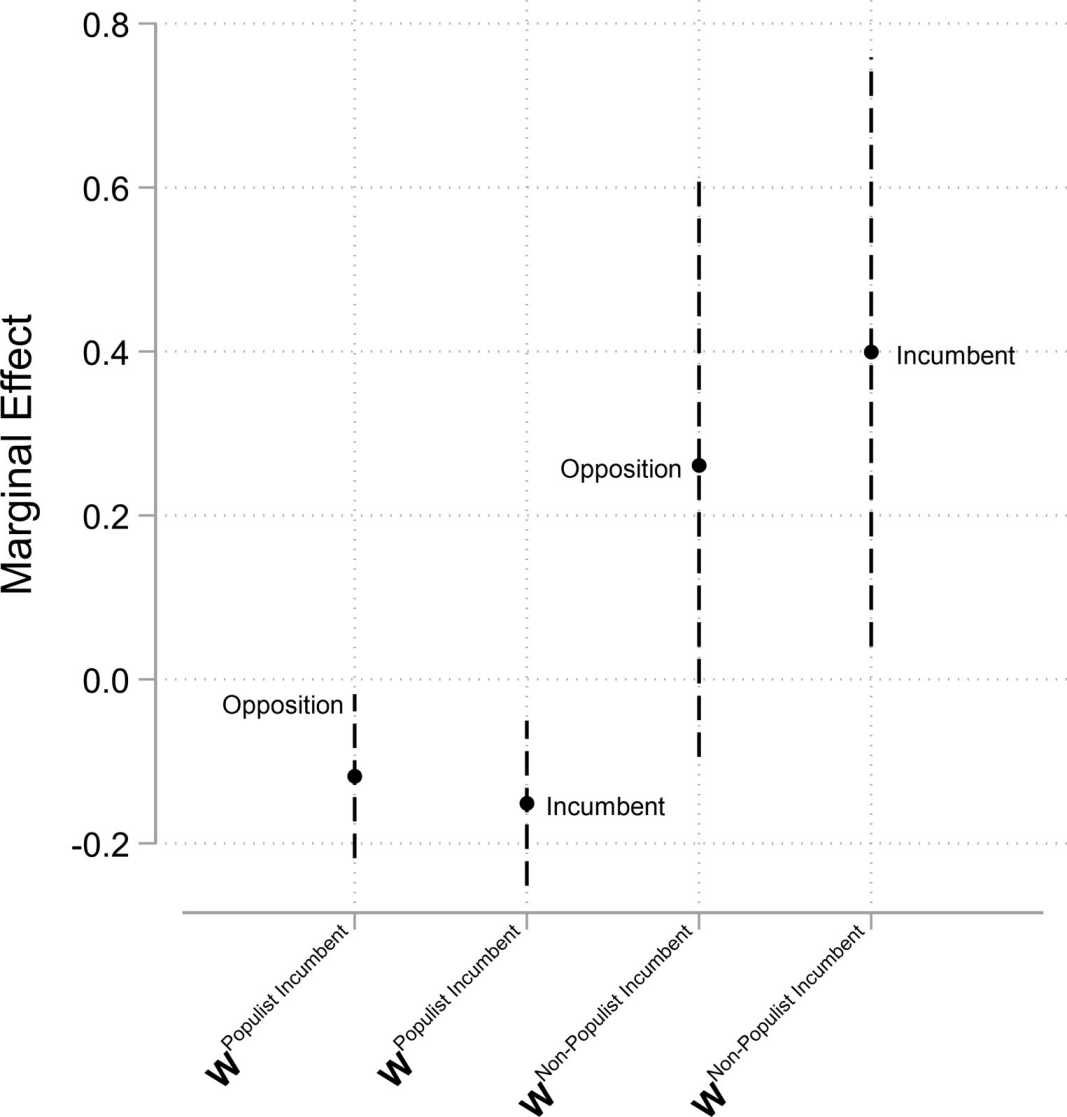
Asymptotic long-term effects. *Notes*. Point estimates pertain to average marginal effects. Dashed lines signify 95 percent confidence intervals. Estimates based on Model 3.

The inclusion of the temporally lagged dependent variable as well as the choice not to row-standardize the underlying matrices complicate the interpretation of the results presented in Table 1. We, therefore, calculate substantive quantities of interest and present these in Figs 1 and 2: the former plots the short-term diffusion effects, while the latter summarizes the asymptotic long-term diffusion effects. For the short-term effects, we multiply a spatial lag’s coefficient by the average number of neighbors. For the long-term effects of spatial lags, we implement the modifications suggested in [36].

The results of Model 1 are consistent with our “policy backlash” hypothesis: domestic parties tend to shift their policy positions *away* from foreign populist incumbents’ positions, all else equal, as indicated by the negative and significant coefficient on the ***Wy***^*Populist Incumbent*^ variable. Substantively, a one-unit change in foreign populist incumbents’ mean lagged positions prompts the receiving party at home to shift by 0.033 units (standard error of 0.0157) in the opposite direction in the short term (calculated by multiplying the coefficient of ***Wy***^*Populist Incumbent*^ in Model 1 by the average number of foreign populist incumbents, which is 2.55). Additional analyses in which we disaggregate the reactions of populist and non-populist receiving parties to foreign populists in power (S12 Table in S1 Appendix) show that this backlash is driven by receiving parties that are not themselves populists (i.e., the vast majority of receiving parties in our sample), while populist parties actually emulate each other across borders. The backlash response thus appears critical in counteracting the “mainstreaming” of populist incumbents’ policies, limiting their spread to other types of parties abroad.

In Model 2, we contrast Model 1 with parties’ responses to governing parties that are not populist. The positive estimates on the ***Wy***^*Non-Populist Incumbent*^ and also ***Wy***^*Domestic*^ variable replicate previous findings, which show that parties learn from foreign non-populist incumbents and from other parties in their own system [8, 29]. Substantively, a one-unit change in the mean position of all foreign non-populist incumbents along the left-right scale prompts a short-term 0.079-unit change (standard error of 0.0157) in a receiving party’s position in the same direction (calculated by multiplying the coefficient on the ***Wy***^Non-Populist Incumbent^ variable in Model 1 by the average number of foreign non-populist incumbents, which is 23.93).

In Model 3, we estimate the reactions to foreign populist and non-populist governing parties jointly and we examine how receiving parties in government (rather than in opposition) differ in their response to these two types of incumbents. Figs 1 and 2 display the predicted short- and long-term effects based on Model 3. We find that both types of domestic parties display a statistically significant short-term backlash to foreign populist incumbents: a one-unit shift in the mean foreign populist incumbent position predicts a shift of 0.025 to 0.030 units (standard error of 0.0157 for both estimates) in the opposite direction for each type of domestic party. The short-term reaction to non-populist incumbents abroad, by contrast, is positive, but differs slightly between governing and opposition parties (Fig 1): in response to a mean one-unit shift in foreign non-populist incumbent positions, governing parties shift their positions by roughly 0.09 units (standard error of 0.0157) in the same direction, a statistically significant effect. Opposition parties’ predicted response is smaller and does not reach statistical significance.

The long-term effects displayed in Fig 2 are considerably larger. A mean one-unit position shift of foreign populist incumbents provokes a backlash from both governing and opposition parties, with predicted long-term shifts of 0.10 (standard error of 0.0156) to 0.15 units (standard error of 0.0155) in the opposite direction. Foreign non-populist incumbents, on the other hand, prompt long-term shifts by domestic governing parties of roughly 0.40 units (standard error of 0.0144) in the same direction, a statistically significant effect. Again, as explained above, parties’ long-term distancing response to foreign populist incumbents on a per-party basis is stronger than their positive response to foreign non-populist incumbents.

### Robustness checks

In the S1 Appendix, we report additional analyses that further substantiate our argument and results. S1 Table in S1 Appendix controls for additional economic influences. S2 Table in S1 Appendix presents analyses that account for the possibility that the Cold War or states’ EU membership may influence programmatic policy diffusion. In S3 Table in S1 Appendix, we omit parties with high levels of uncertainty for their measured policy position estimates, while S4 Table in S1 Appendix distinguishes between foreign populists who govern alone versus those who govern in a coalition. In S5 Table in S1 Appendix, we introduce the distinction between left- and right-wing foreign populist incumbents. S6-S8 Tables in S1 Appendix extend the analysis from the left-right dimension to parties’ positions on EU integration, the economy, and culture. In S9 Table in S1 Appendix, we explore different cut-off points for capturing populist incumbents abroad. S10 Table in S1 Appendix limits the time period of our study to 1995–2017 to address the concern that European populist parties increasingly joined governments only as of the mid-1990s. For a similar reason, S11 Table in S1 Appendix employs the time-variant *PopuList* data set. S12 Table in S1 Appendix considers whether populist receiver parties behave differently from other receiver parties. S13 Table in S1 Appendix is based on maximum likelihood (instead of OLS). Finally, S14 Table in S1 Appendix presents the results from a fully specified spatial-lag interaction approach for which we created multiple different spatial variables. These variables were designed to estimate effects for eight different combinations of sending and receiving parties.

## Discussion

Our work makes several contributions. We offer the first large-scale comparative study testing how populist parties’ government participation affects the spread of their policies to parties abroad. Contrary to earlier research which highlights contagion effects associated with populists’ electoral success [37, 38], we argue and empirically document a ‘populist backlash effect’ that obtains when populists actually enter government. This effect arises, we argue, because participation in government exposes the unique governing dilemmas arising from populist parties’ programs, prompting parties in other countries to systematically distance their policies from those of foreign populist incumbents. For the populism literature, which has focused on explaining the rise in populism [1, 37, 38], our study demonstrates that an international policy backlash occurs when populists enter government. Our study thereby extends recent work stressing that populists’ success—and their subsequent problems—can exert countervailing effects abroad [39–42]. More generally, our findings suggest possible long term “thermostatic effect” with respect to the cross-national diffusion of populist policies. As populist parties’ popular appeal has risen across Western democracies in the 21^st^ century, in response to economic globalization along with sharpening debates over “culture war” issues such as race, immigration, and multiculturalism, populist parties have more frequently entered government. Yet, our findings imply that political parties respond to the distinctive and visible difficulties encountered by populist incumbents abroad by employing a heuristic of distancing their own positions from these negative precedents.

We also redress the policy diffusion literature’s longstanding focus on “positive learning” [see 43, 44], which focuses on the positive transnational diffusion of parties’ programmatic stances. We document instead that parties also respond to negative precedents, i.e., challenging tradeoffs and difficulties foreign populist incumbents confront, by consciously distancing their own positions from these incumbents. That is, our study documents that populists’ highly visible governing dilemmas provoke a backlash among parties in other countries, which undercuts the cross-national spread and “mainstreaming” of populist policies. These findings are timely given the increasing rate at which populists have entered government in recent years.

Our results open interesting avenues for future research. First, our theory posits that parties distance themselves from foreign populist incumbents to enhance their competitiveness, and future qualitative research on cross-national linkages between parties’ organizations and electoral campaigns can further illuminate this process. Second, future work might extend our focus on party responses along the positional dimensions of Left-Right, EU integration, economics, and culture, to consider whether parties additionally adjust the tone of their rhetoric in response to foreign populist incumbents’ anti-system, anti-elite stances. Such a study might analyze pro- or anti-elite tones, for instance, through the language of MPs in parliamentary debates or in party press releases. Third, additional research might explore heterogeneity in the backlash response among different types of receiving parties. While we have analyzed some characteristics of receiver parties–for example, whether they are in government or in opposition and whether they are themselves populists–it is possible that parties on the left or right, or “dominant” and “challenger” parties [2], respond differently to foreign populists in government. Fourth, the implications of the backlash effects we identify require further study. While they may lead to more moderate policy competition in some countries, very strong backlash effects could instead intensify polarization [45]. There is also an interesting question about whether the backlash effects that we identify among political parties is replicated in the media discourse. Fifth, there is evidence that populist ideologies diffuse positively in the domestic context [4], which poses the question why there is positive learning at the domestic level and a backlash response internationally. One explanation that will need further study may be that when populist parties enter the *electoral arena* as *challengers*, other parties emulate them. However, when their rise to the *government arena* exposes the governing dilemmas inherent in their programmatic stances, these positive effects reverse. Finally, our argument is based on two interrelated mechanisms: populists’ inability to successfully address pressing national problems and their poor track record of implementing their policy promises. Qualitative work or more disaggregated data could help distinguish between these mechanisms, thus illuminating the specific drivers of the populist backlash effect. No matter how scholars choose to extend our study, our findings highlight that it is important to account for both positive *and* negative backlash diffusion effects.

## Supporting information

S1 Appendix This document contains additional information associated with the manuscript. This includes robustness checks pertaining to economic influences, Cold War and EU membership, imprecise party positions, government types, left- and right-wing sender parties, alternative data on party positions, different cut-off points for populism, a different time period, alternative, time-variant data on populism, populist incumbent receivers of information, a different estimation technique, and a multiple-spatial lag model.
(DOCX)
