# Supplementary material for: Backlash policy diffusion to populists in power
Source: PLoS One. 2022 Sep 23;17(9):e0273951. doi: 10.1371/journal.pone.0273951 (PMC9506617; doi:10.1371/journal.pone.0273951)
Supplement: S1 Appendix — This includes robustness checks pertaining to economic influences, Cold War and EU membership, imprecise party positions, government types, left- and right-wing sender parties, alternative data on party positions, different cut-off points for populism, a different time period, alternative, time-variant data on populism, populist incumbent receivers of information, a different estimation technique, and a multiple-spatial lag model. (DOCX) [file pone.0273951.s001.docx]

**Supporting Information for**

Backlash Policy Diffusion to Populists in Power

### James F. Adams, Tobias Böhmelt, Lawrence Ezrow, and Petra Schleiter

Corresponding author:

### Tobias Böhmelt

### Department of Government

### University of Essex

### Colchester CO4 3SQ

### United Kingdom

[tbohmelt@essex.ac.uk](mailto:tbohmelt@essex.ac.uk)

**This file includes:**

Supplementary text

Tables S1 to S13

SI References

**Supporting Information**

The Supporting Information provides a set of additional analyses that further support the argument and findings of our paper. These include:

- Table S1 **controls for economic influences**.
- Table S2 considers influences from the **Cold War** and **EU membership**.
- Table S3 **omits** all parties for which **policy positions are likely to be too imprecise**.
- In Table S4, we distinguish between **single-party** and **coalition** sender parties.
- We also distinguish between **left** and **right-wing populist** sender parties (Table S5).
- In Tables S6-S8, we replace the general left-right position of parties by their views on **EU integration**, the **economy**, and **culture**.
- We employ **different cut-off points** to capture populist incumbents abroad (Table S9).
- The temporal scope of the analysis is limited to the **post-1994 period** (Table S10).
- We use **alternative, time-variant data on populism** (Table S11).
- Accounting for **populist incumbent receivers** of information (Table S12).
- The core model using **maximum likelihood estimation** (Table S13).
- **A multiple-spatial lag model** comprising spatial variables for all possible scenarios (Table S14).

**Controlling for Economic Influences**

Williams (2015), Williams and Whitten (2015), and Williams, Seki, and Whitten (2016) use a broader set of alternative predictors including economic variables. We re-estimated our main model, focusing on the distancing from foreign populists, with controls for GDP growth, unemployment, and inflation (all are temporally lagged by one year), which are taken from the World Bank Development Indicators. First, inflation is measured by the consumer price index, reflecting the annual percentage change in the cost to the average consumer of acquiring a basket of goods and services that may be fixed or changed at specified intervals, such as yearly. Second, the World Bank defines economic growth as the annual percentage growth rate of GDP at market prices based on constant local currency. Aggregates are based on constant 2010 US dollars. Finally, unemployment refers to the share of the labor force that is without work but available for and seeking employment. Table S1 summarizes our main model incorporating these additional economic controls. Our core result is robust, while the economic controls do not seem to shape parties’ policy positions in substantive ways. The item capturing unemployment is the only exception here: the higher the unemployment in a country, the more parties shift to the right.

TABLE S1

POLICY DIFFUSION POPULIST BACKLASH – ECONOMIC INFLUENCES

|  | Model A1 |
| --- | --- |
| Lagged Party Position | 0.729 |
|  | (0.014)*** |
| Lagged Median Voter | 0.080 |
|  | (0.234) |
| Lagged Economic Globalization | 0.012 |
|  | (0.016) |
| Lag Median Voter * Lagged Economic Globalization | -0.002 |
|  | (0.003) |
| GDP Growth | 0.003 |
|  | (0.004) |
| Inflation | -0.002 |
|  | (0.005) |
| Unemployment | 0.007 |
|  | (0.003)** |
| **W**y^Domestic^ | 0.004 |
|  | (0.001)*** |
| **W**y^Populist Incumbent^ | -0.016 |
|  | (0.005)*** |
| Observations | 2,779 |
| Year and Party FEs | Yes |
| R^2^ | 0.843 |
| Prob > F | 0.000 |

*Notes.* Table entries are coefficients; standard errors in parentheses; constant as well as year and party fixed effects included in all models, but omitted from presentation; all explanatory variables are one-year lags; the spatial lags capture parties’ positions of the year before the last election.

* p<0.10; ** p<0.05; *** p<0.01

**Controlling for Changes in the International Political Context**

System-wide shocks that affect all parties in our sample may well shape what issues parties perceive as policy priorities and, thereby, alter their positions. The year-fixed effects we include address this issue to a large degree. That said, we also decided to examine two other influences: the end of the Cold War (which is constructed as a dummy variable that receives the value of 1 until 1991, 0 otherwise) and membership in the European Union (EU; coded as a binary variable with a value of 1 if a party’s country of origin was a member of the EU, 0 otherwise). As Table S2 shows, adding *Cold War* and *EU Membership* does not alter the substance of our main finding. With regards to the added variables, though, the coefficient estimate of the former is negatively signed and statistically significant, which suggests that parties were more left-wing during the Cold War. The coefficient for the effect of EU membership is positively signed, but statistically insignificant.

**Excluding Cases with Highly Uncertain Party Positions**

Benoit, Laver, and Mikhaylov (2009) estimate the uncertainty surrounding the party-policy position data of the Comparative Manifesto Project (CMP), namely, the data that we use for our dependent variable and for the construction of the spatial lags. By simulating the data’s underlying error-generating processes via bootstrapping analyses of coded quasi-sentences, Benoit, Laver, and Mikhaylov (2009) then provide precise levels of nonsystematic errors for the left-right party position (among other variables in the CMP). We assessed the uncertainty surrounding each party’s policy position using the left-right standard error estimate in Benoit, Laver, and Mikhaylov (2009). We then omitted all observations from the sample and the corresponding weighting matrices if their standard error estimate was above the 75^th^ percentile of the standard-error variable’s distribution. Table S3 summarizes the findings – our core result remains basically unchanged.

TABLE S2

POLICY DIFFUSION POPULIST BACKLASH – SYSTEMIC INFLUENCES

|  | Model A2 |
| --- | --- |
| Lagged Party Position | 0.779 |
|  | (0.010)*** |
| Lagged Median Voter | 0.191 |
|  | (0.008)* |
| Lagged Economic Globalization | 0.012 |
|  | (0.008) |
| Lag Median Voter * Lagged Economic Globalization | -0.003 |
|  | (0.002)* |
| Cold War | -0.333 |
|  | (0.137)** |
| EU Member | 0.020 |
|  | (0.046) |
| **W**y^Domestic^ | 0.003 |
|  | (0.001)*** |
| **W**y^Populist Incumbent^ | -0.013 |
|  | (0.004)*** |
| Observations | 4,049 |
| Year and Party FEs | Yes |
| R^2^ | 0.872 |
| Prob > F | 0.000 |

*Notes.* Table entries are coefficients; standard errors in parentheses; constant as well as year and party fixed effects included in all models, but omitted from presentation; all explanatory variables are one-year lags; the spatial lags capture parties’ positions of the year before the last election.

* p<0.10; ** p<0.05; *** p<0.01

TABLE S3

POLICY DIFFUSION POPULIST BACKLASH – OMITTING UNCERTAIN PARTY POSITIONS

|  | Model A3 |
| --- | --- |
| Lagged Party Position | 0.750 |
|  | (0.014)*** |
| Lagged Median Voter | 0.533 |
|  | (0.161)*** |
| Lagged Economic Globalization | 0.037 |
|  | (0.012)*** |
| Lag Median Voter * Lagged Economic Globalization | -0.008 |
|  | (0.002)*** |
| **W**y^Domestic^ | 0.005 |
|  | (0.001)*** |
| **W**y^Populist Incumbent^ | -0.021 |
|  | (0.006)*** |
| Observations | 2,184 |
| Year and Party FEs | Yes |
| R^2^ | 0.859 |
| Prob > F | 0.000 |

*Notes.* Table entries are coefficients; standard errors in parentheses; constant as well as year and party fixed effects included in all models, but omitted from presentation; all explanatory variables are one-year lags; the spatial lags capture parties’ positions of the year before the last election.

* p<0.10; ** p<0.05; *** p<0.01

**Single-Party vs. Coalition Governments**

We also distinguish between foreign populists in single-party governments and those in coalitions. Observing populist incumbents in either context may have a different effect on parties at home. That is, the incumbency status could, in principle, imply two different observable implications regarding “foreign populist backlash.” In coalition, the linkage between the tensions in populists’ programmatic positions, government performance and subsequent electoral results may be diluted by populist parties’ status as a (typically junior) coalition partner. When populists rule in single-party governments, those linkages may be more direct. In order to evaluate the effects by the type of government, we revised the foreign-party spatial lags accordingly. The data on governmental type are reported by Döring and Manow (2012).

TABLE S4

POLICY DIFFUSION POPULIST BACKLASH – SINGLE-PARTY VS. COALITIONS

|  | Model A4 |
| --- | --- |
| Lagged Party Position | 0.779 |
|  | (0.010)*** |
| Lagged Median Voter | 0.182 |
|  | (0.114) |
| Lagged Economic Globalization | 0.012 |
|  | (0.008) |
| Lag Median Voter * Lagged Economic Globalization | -0.002 |
|  | (0.002) |
| **W**y^Domestic^ | 0.003 |
|  | (0.001)*** |
| **W**y^Populist Incumbent – Single-Party Government^ | 0.041 |
|  | (0.064) |
| **W**y^Populist Incumbent – Coalition^ | -0.013 |
|  | (0.004)*** |
| Observations | 4,049 |
| Year and Party FEs | Yes |
| R^2^ | 0.872 |
| Prob > F | 0.000 |

*Notes.* Table entries are coefficients; standard errors in parentheses; constant as well as year and party fixed effects included in all models, but omitted from presentation; all explanatory variables are one-year lags; the spatial lags capture parties’ positions of the year before the last election.

* p<0.10; ** p<0.05; *** p<0.01

The findings suggest that parties are particularly likely to distance themselves from foreign populist parties if they were part of a coalition government. This result may be due to the fact that there are (1) a limited number of cases with fewer single party governments than coalition ones, and (2) that both single-party-majority and single-party-minority governments are clustered in the former category. Indeed, as Akkerman and de Lange (2012) note, radical-right populists are often junior coalition partners.

**Distinction between Left- and Right-Wing Populists**

We also estimated the conditioning effect of ideology. Although populism is largely seen as orthogonal to left-right ideological positions (Rooduijn and Akkerman 2017; Otjes and Louwerse 2015; Akkerman et al. 2017; Huber and Ruth 2017; Huber and Schimpf 2017; Taggart 2002; Forchtner and Kølvraa 2015) and, indeed, the main characteristics of populism are the same across left-right ideologies (Mudde 2004), scholars increasingly analyze the relationship between these dimensions (Otjes and Louwerse 2015; Akkerman et al. 2017; Huber and Ruth 2017; Huber and Schimpf 2017). The theoretical rationale behind a moderating influence may be that right-wing populists define and see “the good people” and “corrupt elites” somewhat differently than left-wing populists: the former have a “strong cultural and nativist aspect” in their discourse, while the latter’s discourse is mainly defined in economic terms (Forchtner and Kølvraa 2015: 199; Huber and Schimpf 2017). We use the CMP data to capture the general ideology of parties and distinguish between left-wing and right-wing populist parties abroad. Table S5 shows that ideology is unlikely to matter for the populist backlash pattern. Specifically, whether a populist party abroad is left- or right-wing, parties at home seek to distance themselves.

TABLE S5

POLICY DIFFUSION POPULIST BACKLASH – LEFT AND RIGHT-WING POPULISTS

|  | Model A5 |
| --- | --- |
| Lagged Party Position | 0.778 |
|  | (0.010)*** |
| Lagged Median Voter | 0.174 |
|  | (0.114) |
| Lagged Economic Globalization | 0.012 |
|  | (0.008) |
| Lag Median Voter * Lagged Economic Globalization | -0.002 |
|  | (0.002) |
| **W**y^Domestic^ | 0.003 |
|  | (0.001)*** |
| **W**y^Populist Incumbent – Left-Wing^ | -0.021 |
|  | (0.008)** |
| **W**y^Populist Incumbent – Right-Wing^ | -0.011 |
|  | (0.005)** |
| Observations | 4,049 |
| Year and Party FEs | Yes |
| R^2^ | 0.872 |
| Prob > F | 0.000 |

*Notes.* Table entries are coefficients; standard errors in parentheses; constant as well as year and party fixed effects included in all models, but omitted from presentation; all explanatory variables are one-year lags; the spatial lags capture parties’ positions of the year before the last election.

* p<0.10; ** p<0.05; *** p<0.01

**Alternative Policy Dimensions: EU Integration, Culture, Economics**

As discussed in the main text, left-right ideological structuring in Eastern Europe may differ from that in Western Europe (Evans and Whitefield 1993) as well as across countries and time (Evans and Whitefield 1998; Harbers, De Vries, and Steenbergen 2012; Linzer 2008; Markowski 1997). What is more, the literature increasingly emphasizes the importance of a two-dimensional political space with regards to voter appeal. Our main analysis focuses on the left-right scale and, thus, a one-dimensional issue space. In order to fully account for the multidimensionality of the policy space occupied by political parties, we also examined several other more focused policy positions of parties: EU integration, culture, and economic party positions.

TABLE S6

ECONOMIC POLICY DIMENSION COMPONENTS

| Per401: | Free Market Economy |
| --- | --- |
| Per402: | Incentives: Positive |
| Per403: | Market Regulation |
| Per404: | Economic Planning |
| Per405: | Corporatism/Mixed Economy |
| Per406: | Protectionism: Positive |
| Per407: | Protectionism: Negative |
| Per409: | Keynesian Demand Management |
| Per412: | Controlled economy |
| Per413: | Nationalization |
| Per414: | Economic Orthodoxy |
| Per415: | Marxist Analysis |
| Per416: | Anti-Growth Economy: Positive |
| Per504: | Welfare State Expansion |
| Per505: | Welfare State Limitation |

First, we returned to the CMP data (Budge et al. 2001; Klingemann et al. 2006; Volkens et al. 2015) and coded parties’ positions on EU integration: following the operationalization of the “traditional” left-right position of parties, we concentrate on positive and negative statements in manifestos to create a variable capturing parties’ positions toward EU integration on a scale of 1 to 10, with higher values indicating more positive positions regarding integration. We then use this variable as an alternative dependent variable modifying the temporally lagged dependent variable as well as the spatial lag of our main model accordingly.

Second, we measure party positions on the economic dimension also in light of the coding scheme from the Comparative Manifesto Project website.^[[1]](#footnote-1)^ Specifically, a party’s position on the economic dimension is constructed via:

(per401 + per402 + per407 + per414 + per505) - (per403 + per404 + per405 + per406 + per409 + per412 + per413 + per415 + per416 + per504),

with the substantive interpretations summarized in Table S6. The resulting variable follows a scale from 1 to 10 with lower values standing for a more state-based economy and higher values pertaining to a more market-based system.

Third, each party’s position on the cultural dimension was constructed from the following formula from the Comparative Manifesto Project website:^[[2]](#footnote-2)^

(per104 + per109 + per601 + per605 + per608) – (per105 + per106 + per107 + per501 + per503 + per602 + per604 + per607 + per705),

with the substantive interpretations summarized in Table S7. The resulting variable follows a scale from 1 to 10 with lower values standing for a more progressive society and higher values pertaining to a more conservative system.

TABLE S7

CULTURE POLICY DIMENSION COMPONENTS

| Per104: | Military: Positive |
| --- | --- |
| Per105: | Military: Negative |
| Per106: | Peace |
| Per107: | Internationalism: Positive |
| Per109: | Internationalism: Negative |
| Per501: | Environmental Protection |
| Per503: | Equality: Positive |
| Per601: | National Way of Life Positive |
| Per602: | National Way of Life: Negative |
| Per603: | Traditional Morality: Positive |
| Per604: | Traditional Morality: Negative |
| Per605: | Law and Order Positive |
| Per607: | Multiculturalism: Positive |
| Per608: | Multiculturalism: Negative |
| Per705: | Underprivileged Minority Groups |

Table S8 summarizes the main results using the alternative dependent variables. Note that we now omit the substantive control variables as these are only appropriate for the general left-right dimension, although including the items for the median voter, globalization, and their interaction does not affect the substance of this robustness check. Models S6-S8 demonstrate that the populist backlash effect is robust across alternative policy dimensions, as all spatial lags are negatively signed and significant. However, the substance of the effect does vary. The effect size decreases considerably for the EU integration dimension, but is similar to what we report in the main text when focusing on the economic or culture policy positions of parties.

TABLE S8

POLICY DIFFUSION POPULIST BACKLASH – ALTERNATIVE POLICY DIMENSIONS

|  | Model A6 | Model A7 | Model A8 |
| --- | --- | --- | --- |
|  | EU Integration | Economy | Culture |
| Lagged Party Position | 0.010 | 0.106 | 0.094 |
|  | (0.002)*** | (0.006)*** | (0.006)*** |
| **W**y^Domestic^ | -0.000 | 0.007 | 0.005 |
|  | (0.000) | (0.001)*** | (0.001)*** |
| **W**y^Populist Incumbent^ | -0.004 | -0.013 | -0.014 |
|  | (0.002)** | (0.005)*** | (0.004)*** |
| Observations | 3,514 | 3,514 | 3,514 |
| Year and Party Fes | Yes | Yes | Yes |
| R^2^ | 0.052 | 0.211 | 0.215 |
| Prob > F | 0.000 | 0.000 | 0.000 |

*Notes.* Table entries are coefficients; standard errors in parentheses; constant as well as year and party fixed effects included in all models, but omitted from presentation; all explanatory variables are one-year lags; the spatial lags capture parties’ positions of the year before the last election.

* p<0.10; ** p<0.05; *** p<0.01

**Alternative Thresholds to Define Populist Parties**

For the main analysis, we employ Meijers and Zaslove’s (2021) POPPA data, which define parties as populist on a score ranging between (a party is not at all populist) and 10 (a party is very populist). Our spatial lags in the main text are based on a cut-off point of 5, i.e., we define parties as populist if they score a value higher than 5. We explore the robustness of our results by using instead the cut-off points of 4, 4.5, 5.5, and 6. This robustness check is summarized in Table S9.

TABLE S9

POLICY DIFFUSION POPULIST BACKLASH – ALTERNATIVE CUT-OFF POINTS

|  | Model A9 | Model A10 | Model A11 | Model A12 |
| --- | --- | --- | --- | --- |
|  | Cut-Off 4 | Cut-Off 4.5 | Cut-Off 5.5 | Cut-Off 6 |
| Lagged Party Position | 0.779 | 0.779 | 0.779 | 0.779 |
|  | (0.010)*** | (0.010)*** | (0.010)*** | (0.010)*** |
| Lagged Median Voter | 0.209 | 0.195 | 0.188 | 0.207 |
|  | (0.114)* | (0.113)* | (0.114)* | (0.114)* |
| Lagged Economic Globalization | 0.015 | 0.014 | 0.013 | 0.015 |
|  | (0.008)* | (0.008)* | (0.008) | (0.008)* |
| Lag Median Voter * | -0.003 | -0.003 | -0.003 | -0.003 |
| Lagged Economic Globalization | (0.002)* | (0.002)* | (0.002)* | (0.002)* |
| **W**y^Domestic^ | 0.003 | 0.003 | 0.003 | 0.003 |
|  | (0.001)*** | (0.001)*** | (0.001)*** | (0.001)*** |
| **W**y^Populist Incumbent^ | -0.004 | -0.014 | -0.011 | -0.004 |
|  | (0.004) | (0.005)*** | (0.004)*** | (0.005) |
| Observations | 4,049 | 4,049 | 4,049 | 4,049 |
| Year and Party Fes | Yes | Yes | Yes | Yes |
| R^2^ | 0.872 | 0.872 | 0.872 | 0.872 |
| Prob > F | 0.000 | 0.000 | 0.000 | 0.000 |

*Notes.* Table entries are coefficients; standard errors in parentheses; constant as well as year and party fixed effects included in all models, but omitted from presentation; all explanatory variables are one-year lags; the spatial lags capture parties’ positions of the year before the last election.

* p<0.10; ** p<0.05; *** p<0.01

The main finding is robust when altering the cut-off point to define populist parties to either 4.5 or 5.5. However, the spatial lag becomes insignificant when moving to 4 or 6 on *Populism Score*. The reason for this is twofold. On one hand, employing a threshold value of 4 comprises too many parties that are not openly or clearly populist anymore. Distancing from these, in turn, becomes more difficult, leading to the overall insignificance of the spatial lag. On the other hand, using a cut-off point of 6 focuses on the extreme populist cases in the data, but there is not a sufficiently high number of data points for the spatial lag to produce meaningful results. Our whole data sample comprises 4,049 party-years, of which 1,405 only pertain to incumbents. A cut-off point of 5 pertains to 461 populist incumbent-years and only 16 parties, using the value of 6 instead lowers this to 434 party-years and only 12 parties.

TABLE S10

POLICY DIFFUSION POPULIST BACKLASH – POST-1994 PERIOD

|  | Model A13 |
| --- | --- |
| Lagged Party Position | 0.757 |
|  | (0.013)*** |
| Lagged Median Voter | -0.246 |
|  | (0.240) |
| Lagged Economic Globalization | -0.017 |
|  | (0.017) |
| Lag Median Voter * Lagged Economic Globalization | 0.003 |
|  | (0.003) |
| **W**y^Domestic^ | 0.002 |
|  | (0.001)* |
| **W**y^Populist Incumbent^ | -0.016 |
|  | (0.004)*** |
| Observations | 2,777 |
| Year and Party Fes | Yes |
| R^2^ | 0.862 |
| Prob > F | 0.000 |

*Notes.* Table entries are coefficients; standard errors in parentheses; constant as well as year and party fixed effects included, but omitted from presentation; all explanatory variables are one-year lags; the spatial lags capture parties’ positions of the year before the last election.

* p<0.10; ** p<0.05; *** p<0.01

**Limiting the Temporal Scope of the Analysis**

We focus on the time period 1977 to 2017. However, most populist parties joined governments not before the mid-1990s. In addition, our measure of populist parties is time-invariant, which may give rise to a concern that, for instance, the Swiss People’s Party or the Austrian FPÖ were not populist prior to the mid-1990s. We have therefore re-estimated our main model for the post-1994 period only. The results in Table S10 are qualitatively identical to what we discuss in the main text.

**Alternative Data on Populism**

For identifying populist parties, we use the Populism and Political Parties Expert Survey (POPPA) by Meijers and Zaslove (2021). The large spatial and temporal coverage of this data set is a key advantage, although it is a cross-section of parties in 2017/2018 only. The previous robustness check (Table S11) addresses this shortcoming partly as we restrict the temporal scope. In the following, we present another analysis to address the same concern: we employ alternative, time-variant data on populism. The PopuList data set^[[3]](#footnote-3)^ provides information on European parties from 31 countries that either won at least one seat or 2% of the votes in national parliamentary elections since 1989. The coding of populist parties (binary variable) is based on Mudde (2004) and, according to the codebook of PopuList, parties must “endorse the set of ideas that society is ultimately separated into two homogeneous and antagonistic groups, “the pure people” versus “the corrupt elite,” and which argues that politics should be an expression of the volonté énérale (general will) of the people.” We take this dichotomous variable to construct our matrix of **W**y^Populist Incumbent^. While the PopuList data set provides time-varying information on political parties, the spatio-temporal coverage is limited in that our analysis only covers 1989-2017. All other model specifications remain unaltered. In Model A14, the spatial variable ***W****y^Populist Incumbent^* remains negatively signed and is statistically significant at conventional levels. As a result, we conclude that our analyses and its findings are also robust to alternative, time-varying data on populism.

TABLE S11

POLICY DIFFUSION POPULIST BACKLASH – ALTERNATIVE POPULISM DATA

|  | Model A14 |
| --- | --- |
| Lagged Party Position | 0.758 |
|  | (0.012)*** |
| Lagged Median Voter | -0.180 |
|  | (0.163) |
| Lagged Economic Globalization | -0.006 |
|  | (0.011) |
| Lag Median Voter * Lagged Economic Globalization | 0.002 |
|  | (0.002) |
| **W**y^Domestic^ | 0.003 |
|  | (0.001)*** |
| **W**y^Populist Incumbent^ | -0.031 |
|  | (0.019)* |
| Observations | 3,190 |
| Year and Party FEs | Yes |
| R^2^ | 0.858 |
| Prob > F | 0.000 |

*Notes.* Table entries are coefficients; standard errors in parentheses; constant as well as year and party fixed effects included, but omitted from presentation; all explanatory variables are one-year lags; the spatial lags capture parties’ positions of the year before the last election.

* p<0.10; ** p<0.05; *** p<0.01

TABLE S12

INTERACTION WITH POPULIST RECEIVERS

|  | Model A15 |
| --- | --- |
| Lagged Party Position | 0.779 |
|  | (0.010)*** |
| Lagged Median Voter | 0.183 |
|  | (0.114) |
| Lagged Economic Globalization | 0.013 |
|  | (0.008) |
| Lag Median Voter * Lagged Economic Globalization | -0.002 |
|  | (0.002) |
| **W**y^Domestic^ | 0.003 |
|  | (0.001)*** |
| **W**y^Populist Incumbent^ | -0.010 |
|  | (0.005)** |
| Populist Incumbent | -0.256 |
|  | (0.117)** |
| Populist Incumbent * **W**y^Populist Incumbent^ | 0.017 |
|  | (0.005)*** |
| Observations | 4,049 |
| Year and Party FEs | Yes |
| R^2^ | 0.873 |
| Prob > F | 0.000 |

*Notes.* Table entries are coefficients; standard errors in parentheses; constant as well as year and party fixed effects included, but omitted from presentation; all explanatory variables are one-year lags; the spatial lags capture parties’ positions of the year before the last election.

* p<0.10; ** p<0.05; *** p<0.01

**Accounting for Populist Incumbent Receivers**

Anecdotal evidence suggests that populists copy each other cross-nationally. This gives rise to the question about *which* parties react to populists in government, and whether the backlash we argue for may be conditioned by the party family of the receiving party. To control for this, we isolate the reaction of populist receivers by creating a variable that captures whether receiving parties are coded as populist incumbents themselves (following our criteria introduced in the research design above). In turn, we interact this variable with ***W****y^Populist Incumbent^*. Table S12 presents our findings. On the one hand, the item ***W****y^Populist Incumbent^* remains negatively signed and significant, presenting further evidence for the populist backlash effect. On the other hand, however, the interaction of *Populist Incumbent* with the spatial lag is positively signed and significant at conventional levels: this is in line with the qualitative evidence in the literature that populist parties tend to emulate each other across borders.

**Maximum Likelihood Estimation**

The empirical analyses in the text are based on OLS, following, e.g., Williams (2015; see also Williams and Whitten 2015). However, the problem with OLS models might be that simultaneity bias is present due to the inclusion of the spatial lag, although temporally lagging the variable can address this problem if the first observation in the data is fixed in repeated samples, i.e., there is no stochastic element to it. Maximum likelihood directly corrects for simultaneity bias. In order to ensure that the type of estimator does not affect our findings, we re-estimated our main model using the m-STAR maximum likelihood estimator by Hays, Kachi, and Franzese (2010), Table S13 is thus a maximum-likelihood replication of our main model that we report in the main text: the core effect identified there (using OLS) is almost identical to the results based on maximum likelihood.

TABLE S13

MAXIMUM LIKELIHOOD ESTIMATION

|  | Model A16 |
| --- | --- |
| Lagged Party Position | 0.779 |
|  | (0.010)*** |
| Lagged Median Voter | 0.183 |
|  | (0.109)* |
| Lagged Economic Globalization | 0.013 |
|  | (0.008)* |
| Lag Median Voter * Lagged Economic Globalization | -0.002 |
|  | (0.001)* |
| **W**y^Domestic^ | 0.003 |
|  | (0.001)*** |
| **W**y^Populist Incumbent^ | -0.012 |
|  | (0.004)*** |
| **Wy**^Non-Populist Incumbent^ | 0.003 |
|  | (0.002)* |
| Observations | 4,049 |
| Year and Party FEs | Yes |
| Log Likelihood | -909.559 |
| Prob > χ^2^ | 0.000 |

*Notes.* Table entries are coefficients; standard errors in parentheses; constant as well as year and party fixed effects included, but omitted from presentation; all explanatory variables are one-year lags; the spatial lags capture parties’ positions of the year before the last election.

* p<0.10; ** p<0.05; *** p<0.01

TABLE S14

FULLY SPECIFIED MULTIPLE-SPATIAL LAG MODEL

|  | Model A17 |  |
| --- | --- | --- |
| Lagged Party Position | 0.778 |  |
|  | (0.010)*** |  |
| Lagged Median Voter | 0.191 |  |
|  | (0.114)* |  |
| Lagged Economic Globalization | 0.013 |  |
|  | (0.008)* |  |
| Lag Median Voter * Lagged Economic Globalization | -0.003 | |
|  | (0.002)* | |
| **Wy**^Domestic*Opposition*Non-Populist^ | 0.003 | |
|  | (0.001)*** | |
| **Wy**^Domestic*Opposition*Populist^ | -0.001 | |
|  | (0.003) | |
| **Wy**^Domestic*Incumbent*Non-Populist^ | 0.002 | |
|  | (0.001)* | |
| **Wy**^Domestic*Incumbent*Populist^ | 0.002 | |
|  | (0.003) | |
| **Wy**^Foreign*Opposition*Non-Populist^ | -0.000 | |
|  | (0.000) | |
| **Wy**^Foreign*Opposition*Populist^ | 0.000 | |
|  | (0.000) | |
| **Wy**^Foreign*Incumbent*Non-Populist^ | 0.003 | |
|  | (0.002) | |
| **Wy**^Foreign*Incumbent*Populist^ | -0.012 | |
|  | (0.004)*** | |
| Observations | 4,049 | |
| Year and Party FEs | Yes | |
| R^2^ | 0.873 | |

*Notes.* Table entries are coefficients (and standard errors in parentheses). The constant and year- and party-fixed effects are included, but omitted from the presentation. All explanatory variables are lagged one year. The spatial lags are calculated based on parties’ positions from the year before their last national election.

* p<0.10; ** p<0.05; *** p<0.01

**Fully Specified Multiple-Spatial Lag Model**

We also considered that a spatial-lag interaction can be expressed as a set of differently specified spatial variables and, hence, weight matrices. That is, our full proposition is that domestic/receiving parties react negatively to foreign populists, especially those in government. We thus created multiple different spatial lags for each of these combinations of conditions true about party i (receiving party) and party j (sending party): (i and j same or different country)*(j in government vs opposition)*(j populist or not). This gives us eight different weighting matrices and, as a result, spatial lags, one for each of these binary conditions. For example, the spatial lag ***Wy****^Domestic*Opposition*Non-Populist^* captures two parties from the same country, where the sending party is in opposition and non-populist. In turn, we estimate this one multiple-***W*** model. Table S14 summarizes the corresponding estimation.

In general, our results are identical to what is reported in the main text. ***Wy****^Domestic*Opposition*Non-Populist^* is positively signed and significant, mirroring the effect we identify for ***Wy****^Domestic^* in Table 1 of the manuscript. We also obtain a statistically significant estimate for ***Wy****^Foreign*Incumbent*Populist^*, which captures the scenario of two parties in different countries with the receiving party in government and being characterized as populist. As in the main text, the effect is negatively signed.

**SI References**

Akkerman, Agnes, Cas Mudde, and Andrej Zaslove. 2014. How Populist Are the People? Measuring Populist Attitudes in Voters. *Comparative Political Studies* 47 (9): 1324-1353.

Akkerman, Tjitske, and Sarah de Lange. 2012. “Radical Right Parties in Office: Incumbency Records and the Electoral Cost of Governing.” *Government and Opposition* 47(4): 574-596.

Benoit, Kenneth, Michael Laver, and Slava Mikhaylov. 2009. “Treating Words as Data with Error: Uncertainty in Text Statements of Policy Positions*.” American Journal of Political Science* 53(2): 495-513.

Budge, Ian, Hans-Dieter Klingemann, Andrea Volkens, Judith Bara, and Eric Tanenbaum. 2001. *Mapping Policy Preferences: Estimates for Parties, Electors, and Governments 1945-1998*. Oxford: Oxford University Press.

Döring, Holger, and Philip Manow. 2012. *Parliament and Government Composition Database (ParlGov): An Infrastructure for Empirical Information on Parties, Elections, and Governments in Modern Democracies. Version 12/10 – 15 October 2012*. Available online: <http://parlgov.org/> (accessed on May 10, 2022).

Evans, Geoffrey, and Stephen Whitefield. 1993. “Identifying the Bases of Party Competition in Eastern Europe.” *British Journal of Political Science* 23(4): 521-548.

Evans, Geoffrey, and Stephen Whitefield. 1998. “The Evolution of Left and Right in Post-Soviet Russia.” *Europe-Asia Studies* 50(6): 1023-1042.

Forchtner, Bernhard, and Christoffer Kølvraa. 2015. “The Nature of Nationalism: Populist Radical Right Parties on Countryside and Climate.” *Nature and Culture* 10 (2): 199-224.

Harbers, Imke, Catherine De Vries, and Marco Steenbergen. 2013. “Attitude Variability Among Latin American Publics: How Party System Structuration Affects Left/Right Ideology.” *Comparative Political Studies* 46(8): 947-967.

Hays, Jude, Aya Kachi, and Robert Franzese. 2010. ‘A Spatial Model Incorporating Dynamic, Endogenous, Network Interdependence: A Political Science Application.’ *Statistical Methodology* 7(3): 406-28.

Huber, Robert, and Christian Schimpf. 2016. Friend or Foe? Testing the Influence of Populism on Democratic Quality in Latin America. *Political Studies* 64 (4): 872-889.

Huber, Robert, and Saskia Ruth. 2017. Mind the Gap! Populism, Participation, and Representation in Europe. *Swiss Political Science Review* 23 (4): 462-484.

Klingemann, Hans-Dieter, Andrea Volkens, Judith Bara, Ian Budge, and Michael McDonald. 2006. *Mapping Policy Preferences II: Estimates for Parties, Electors, and Governments in Central and Eastern Europe, European Union, and OECD 1990-2003*. Oxford: Oxford University Press.

Linzer, Drew. 2008. *The Structure of Mass Ideology and Its Consequences for Democratic Governance*. University of California at Los Angeles: PhD Dissertation.

Markowski, Radoslaw. 1997. “Political Parties and Ideological Spaces in East Central Europe.” *Communist and Post-Communist Studies* 30(3): 221-254.

Meijers, Maurits J., and Andrej Zaslove. 2021. “Measuring Populism in Political Parties: Appraisal of a New Approach.” *Comparative Political Studies* 54(2): 372-407.

Mudde, Cas. 2004. The Populist Zeitgeist. *Government and Opposition* 39 (4): 541-563.

Otjes, Simon, and Tom Louwerse. 2015. Populists in Parliament: Comparing Left-Wing and Right-Wing Populism in the Netherlands. *Political Studies* 63 (1): 60-79.

Rooduijn, Matthijs, and Tjitske Akkerman. 2017. Flank Attacks: Populism and Left-Right Radicalism in Western Europe. *Party Politics* 23 (3): 193-204.

Rooduijn, Matthijs, Sarah L. De Lange, and Wouter Van der Brug. 2014. “A Populist Zeitgeist? Programmatic Contagion by Populist Parties in Western Europe.” *Party Politics* 20(4): 563-575.

Taggart, Paul. 2002. Populism and the Pathology of Representative Politics. In: Yves Mény and Yves Surel (eds.). *Democracies and the Populist Challenge*. London: Palgrave, pp. 62-80.

Volkens, Andrea, Pola Lehmann, Nicolas Merz, Sven Regel, and Annika Werner. 2013. *The Manifesto Data Collection. Manifesto Project (MRG/CMP/MARPOR). Version 2013a*. Berlin: Wissenschaftszentrum Berlin für Sozialforschung (WZB).

Williams, Laron. 2015 “It’s All Relative: Spatial Positioning of Parties and Ideological Shifts.” *European Journal of Political Research* 54(1): 141-159.

Williams, Laron, and Guy Whitten. 2015. “Don’t Stand So Close to Me: Spatial Contagion Effects and Party Competition.” *American Journal of Political Science* 59(2): 309-325.

Williams, Laron, Katsunori Seki, and Guy Whitten. 2016. “You’ve Got Some Explaining to Do: The Influence of Economic Conditions and Spatial Competition on Party Strategy.” *Political Science Research and Methods* 4(1): 47-63.

1. See online at: <https://manifesto-project.wzb.eu/information/documents/visualizations>. [↑](#footnote-ref-1)
2. See online at: <https://manifesto-project.wzb.eu/information/documents/visualizations>. [↑](#footnote-ref-2)
3. Available online at: <https://popu-list.org/>. [↑](#footnote-ref-3)
